# Comparative Analyses of Fucoidans from South African Brown Seaweeds That Inhibit Adhesion, Migration, and Long-Term Survival of Colorectal Cancer Cells

**DOI:** 10.3390/md21040203

**Published:** 2023-03-24

**Authors:** Blessing Mabate, Chantal Désirée Daub, Brett Ivan Pletschke, Adrienne Lesley Edkins

**Affiliations:** 1 Enzyme Science Programme (ESP), Department of Biochemistry and Microbiology, Faculty of Science, Rhodes University, Makhanda 6140, South Africa; 2 Biomedical Biotechnology Research Unit (BioBRU), Department of Biochemistry and Microbiology, Rhodes University, Makhanda 6139, South Africa

**Keywords:** cancer, migration, adhesion, fucoidans, human colorectal cancer, *Ecklonia radiata*, *Ecklonia maxima*, *Sargassum elegans*

## Abstract

Human colorectal cancer (CRC) is a recurrent, deadly malignant tumour with a high incidence. The incidence of CRC is of increasing alarm in highly developed countries, as well as in middle to low-income countries, posing a significant global health challenge. Therefore, novel management and prevention strategies are vital in reducing the morbidity and mortality of CRC. Fucoidans from South African seaweeds were hot water extracted and structurally characterised using FTIR, NMR and TGA. The fucoidans were chemically characterised to analyse their composition. In addition, the anti-cancer properties of the fucoidans on human HCT116 colorectal cells were investigated. The effect of fucoidans on HCT116 cell viability was explored using the resazurin assay. Thereafter, the anti-colony formation potential of fucoidans was explored. The potency of fucoidans on the 2D and 3D migration of HCT116 cells was investigated by wound healing assay and spheroid migration assays, respectively. Lastly, the anti-cell adhesion potential of fucoidans on HCT116 cells was also investigated. Our study found that *Ecklonia* sp. Fucoidans had a higher carbohydrate content and lower sulphate content than *Sargassum elegans* and commercial *Fucus vesiculosus* fucoidans. The fucoidans prevented 2D and 3D migration of HCT116 colorectal cancer cells to 80% at a fucoidan concentration of 100 µg/mL. This concentration of fucoidans also significantly inhibited HCT116 cell adhesion by 40%. Moreover, some fucoidan extracts hindered long-term colony formation by HCT116 cancer cells. In summary, the characterised fucoidan extracts demonstrated promising anti-cancer activities in vitro, and this warrants their further analyses in pre-clinical and clinical studies.

## 1. Introduction

Cancer is a complex, multifactorial disease characterised by the uncontrollable growth of abnormal cancerous cells [1]. Cancers may progress to invade and spread to other tissues and organs using the circulatory and lymphatic systems through metastasis [2]. Cancer has one of the highest mortality rates and significantly contributes to lower global life expectancy [3]. The cancer burden globally is expected to increase from 2020 by approximately 47%, translating to about 28.4 million new cases per year by 2040. However, the increased number of cancer cases may be affected by the social-economic status of the global populace. Additionally, the rise may be linked to increased risk factors associated with globalisation and the growing economy [4]. These risk factors may include increased processed food consumption, lack of physical activity, and increasing obesity.

Colorectal cancer (CRC) is the third most diagnosed cancer (accounting for 10% of all cases) and the second most frequent cause of cancer-related deaths (accounting for 9.4% of oncological deaths). Thus, it constitutes a substantial portion of the global cancer burden [4,5]. Treatment strategies include chemotherapy, radiation therapy, surgery, or combination therapies [6]. Although surgical resection of the primary tumour in the early disease stages proves effective, patients may be diagnosed at more advanced stages [7,8]. The indiscriminate toxic effects of chemotherapeutic agents used for CRC treatment result in debilitating side effects and limit therapeutic outcomes [7,9].

With challenges of side effects, affordability, and access to current therapeutic remedies, the search for novel treatment and preventive strategies with minimal adverse effects must proceed urgently. Furthermore, marine bio-products have historically been deemed therapeutic advantages among other bio-compounds [10]. Natural compounds have gained attention over the past decades as these demonstrate targeted specific anti-cancer properties while demonstrating low toxicity [1]. The lower incidences of chronic diseases, such as heart disease, diabetes, and cancer in China and Japan have led researchers to investigate the contents of brown seaweeds, which have been used in their cuisines and medicinal applications [11]. Among the more than 3000 natural products derived from seaweeds, fucoidans have received significant attention for their most promising anti-cancer properties [1,11].

Fucoidan is a heparin-like structured, naturally derived polysaccharide compound present in the cell wall matrix of brown seaweeds [12]. This heterogeneous polysaccharide is predominantly comprised of *l*-fucose with smaller quantities of varying monosaccharides and sulphate, which contribute to its complex structural characteristics and have an unquestionable effect on its broad range of biological activities [1]. These biological activities include anti-oxidant [13], anti-coagulant [14], anti-thrombotic, anti-inflammatory, anti-viral, anti-lipidemic [15], anti-diabetic [16], anti-metastatic and anti-cancer activities [17].

Fucoidans have anti-cancer effects against various cancer cell lines by causing cell cycle arrest [18], inducing apoptosis [9], preventing angiogenesis [9,19], and inhibiting migration and metastasis [1]. As tumour migration is a hallmark of cancers, it is plausible to target this process to alleviate tumour progression. Moreover, fucoidans inhibit metastasis by blocking cell migration and colony formation. Fucoidan isolated from *F. vesiculosus* significantly inhibited the migration of the human colon cancer cell line HT-29 by suppressing PI3k/Akt/mTOR/p70S6K1 [19]. Whereas the treatment of colorectal carcinoma cells, DLD-1 and HCT116, with fucoidan from *Padina boryana,* proved successful in inhibiting colony formation [20]. 

The anti-cancer effect of fucoidan on colon cancer cell lines has been reported primarily using the commercially available *F. vesiculosus* fucoidan. However, the diversity of brown seaweeds is broad, and their bioactivities have been linked to the source of seaweed and its structural and chemical characteristics. Additionally, in addition to the limited literature on fucoidan effects on colon cancers, there is also limited literature available on the biological activities, including the anti-cancer properties of South African seaweed-derived fucoidans. However, the country harbours one of the most extensive coastlines globally, with a rich seaweed biodiversity [21]. The present study characterised fucoidan extracts from native South African brown seaweeds and linked their structural differences to their anti-cancer properties against the HCT116 cell line. 

## 2. Results and Discussion

### 2.1. Fucoidan Yield

The fucoidans in this study were hot water extracted, except for the *F. vesiculosus* fucoidan, which was purchased commercially. Considerable amounts of fucoidans were successfully extracted with an average fucoidan/defatted seaweed dry weight ratio of 5.4, 5.9 and 2.2% for *E. maxima*, *E. radiata* and *S. elegans,* respectively. The resulting yields of the extracted fucoidans were within the expected range (1.1–4.8%) for water extracted fucoidans [22]. 

### 2.2. Structural Analysis of Fucoidans

#### 2.2.1. FTIR Analysis

**Figure 1:**
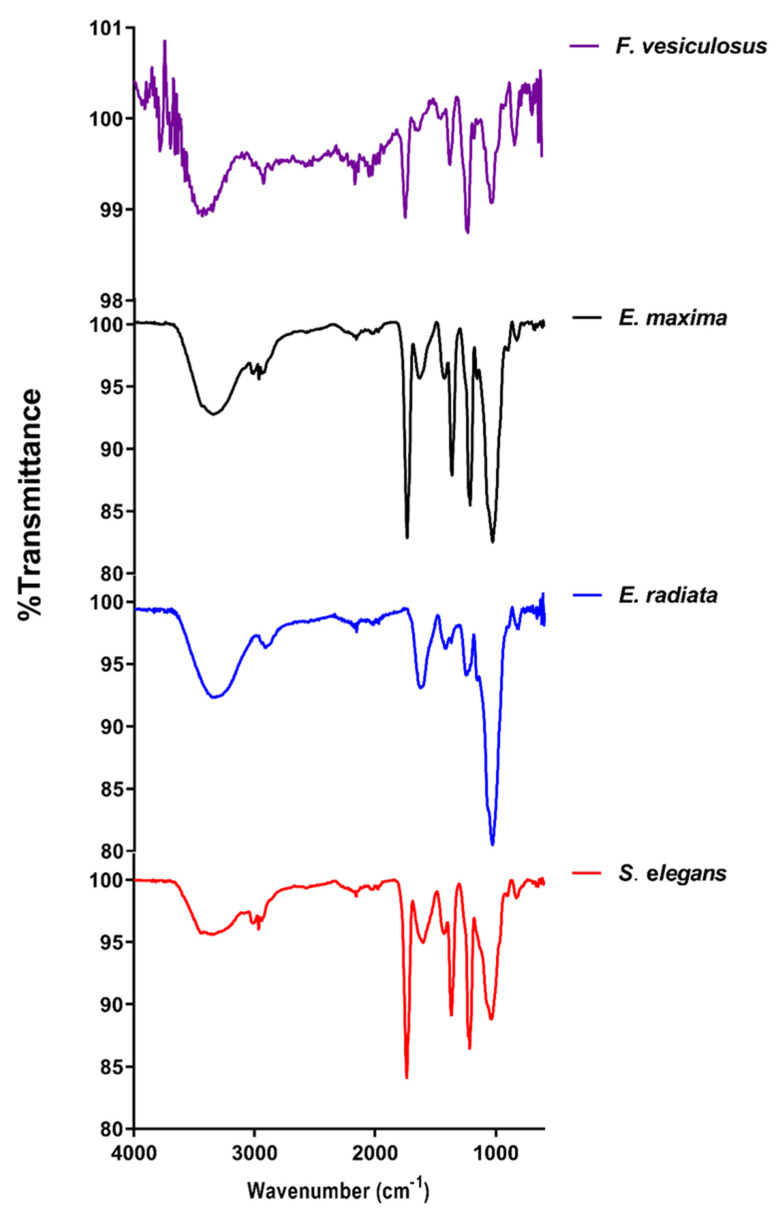
FTIR spectra of the fucoidans under study. The overlaid spectra were obtained from water-extracted fucoidans and the commercial *F. vesiculosus* fucoidan.

Fucoidan extracts were structurally analysed by Fourier-transform infrared spectroscopy (FTIR) (Figure 1) and displayed similar spectra to previously characterised fucoidans [22,23]. All the profiled fucoidans displayed a spectral band between 3500 cm^−1^ and 3200 cm^−1^, characteristic of polysaccharides. This peak is associated with the stretching vibrations of the O-H groups within carbohydrates. The bands in the region 2900 to 3000 cm^−1^ observed in all the fucoidans are assigned to the C-H stretching in the pyranose ring and methyl groups associated with the fucose [24].

Typically, the carbonyl groups and stretching of O-acetyl groups are depicted by the peaks around 1650 cm^−1^ [25]. Additionally, the stretching of the S=O bond linked with sulphate groups is characterised by peaks between 1210 and 1270 cm^−1^ [22]. Stretching vibrations of the glycosidic C–O bonds within the fucoidans structures are represented by peaks close to the wavenumber 1100 cm^−1^ [26]. Furthermore, the peaks at wavenumber 854 cm^−1^ depict sulphate groups on fucoidans linked to carbonyl side chains [13]. This peak at around 854 cm^−1^ was more pronounced for the *F. vesiculosus* commercial fucoidan. This suggested that *F. vesiculosus* fucoidan had a relatively higher sulphate content than the extracted fucoidans (Figure 1).

#### 2.2.2. Proton NMR Analysis of Extracted Fucoidans

**Figure 2:**
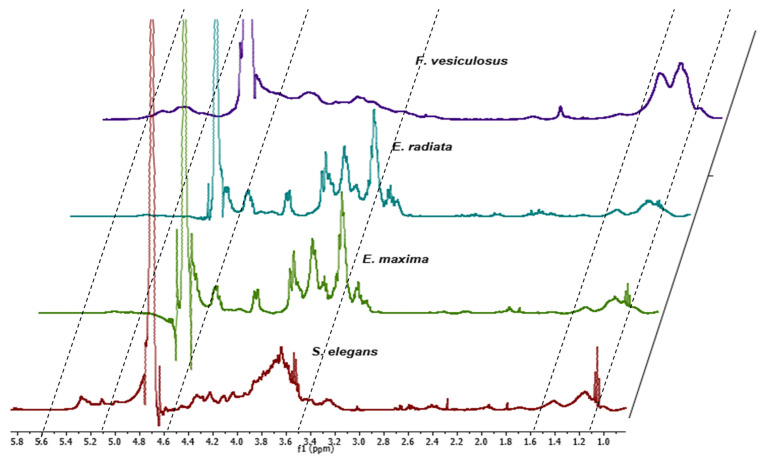
^1^H NMR for the different seaweed fucoidans. Overlaid proton NMR spectra of extracted fucoidans and commercial *F. vesiculosus* fucoidan.

The structural composition of the extracted fucoidans was also elucidated by proton NMR. The fucoidans generally exhibited chemical shifts (Figure 2) similar to several characterised fucoidans [14,27,28]. The chemical shifts in all fucoidans showed peaks at 1.28 ppm and 1.45 ppm suggesting the presence of alternating α (1–3) and α (1–4) linkages of fucose residues linked with sulphates (α-L-Fuc, α-L-Fuc (2-SO_3_^−^) and α-L-Fuc (2,3-diSO_3_^−^) [28]. The *F. vesiculosus* fucoidan displayed relatively more prominent peaks in the 1.28 ppm and 1.45 ppm range, suggesting a higher sulphate content than the extracted fucoidans (Figure 2). A higher sulphate content in the fucoidans may improve their biological activities. 

Vibration bands at 1.45 ppm are assigned to symmetric CH_3_ deformations emanating from methyl proton on C6 of fucose [14]. The peaks at 2.1 ppm are assigned to the H-6 methylated protons of *L*-fucopyranosides [27]. The peaks in the range of 3.5–4.5 ppm are characteristic of the (H2 to H5) ring protons of *L*-fucopyranosides. The exhibited peaks in the ring proton region also suggest variable fucosyl sulphates located at variable glycosidic linkages with varying monosaccharide patterns. The definitive peaks in Figure 2 at 3.3 ppm and 3.7 ppm in the fucoidans suggest the presence of hexoses, including glucose, galactose, and mannose [27]. Our results show that the spectra of *E. maxima* and *E. radiata* fucoidans displayed more pronounced peaks at the 3.3 ppm to 3.7 ppm region than the *F. vesiculosus* and *S. elegans* fucoidans (Figure 2). This could suggest that *Ecklonia* sp. fucoidans have higher sugar content. Furthermore, the extracted fucoidans had limited to negligible uronic acid contamination as there were no chemical shifts in the region around 5.8 ppm (Figure 2) [29].

#### 2.2.3. Thermogravimetric Analysis of Fucoidans

**Figure 3:**
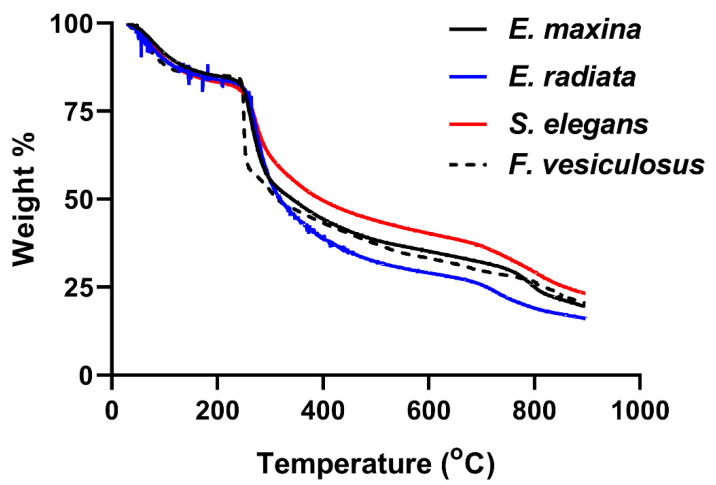
Thermal gravimetric analysis (TGA) analysis of the fucoidan extracts. Superimposed thermograms for the water-extracted fucoidans from seaweeds and commercial *F. vesiculosus* fucoidan.

The TGA decomposition profiles of the fucoidans validated the compounds as polysaccharides, as their decomposition started just above 200 °C (Figure 3), characteristic of the organic polymers [30]. 

The TGA plots of the fucoidans showed about 20% loss in mass at a temperature of 240 °C, associated with the loss in moisture content through evaporation [31] and some volatile matter [32]. The most significant loss of mass (~45%) occurred between 240 °C and 420 °C, which accounted for the arbitrary depolymerisation and decomposition of organic constituents, such as carbohydrates. Notably, *F. vesiculosus* fucoidan depolymerisation and decomposition of organic matter occurred relatively more rapidly than the other fucoidans, as shown by the steeper slope in Figure 3. Its relatively low carbohydrate content may be the reason for this observation (Table 1). Above 420 °C, combustion of carbon black occurred. The remaining residual mass at 600 °C accounted for the ash content, usually containing sulphates, phosphates, and carbonates [33]. The profiles of the extracts were characteristic of previously profiled fucoidan extracts in the literature [34,35]. 

The structural analysis of fucoidans through FTIR, proton NMR and TGA confirmed the integrity of our extracts, as they showed comparable patterns to the commercial *F. vesiculosus* fucoidan. It was also evident that *Ecklonia* sp. fucoidans displayed similar yet unique profiles to the *S. elegans* and *F. vesiculosus* fucoidans. Our observations support the findings of Ermakova and colleagues that diverse seaweed species yield diverse fucoidan structures [36]. These differences may be caused by the survival needs of the seaweeds, influenced by their habitat. Considering the unique profiles observed within the structural analyses of the fucoidans, these were further characterised chemically to assess their composition.

### 2.3. Composition of Fucoidans

The fucoidans were partially characterised chemically by determining their total sugar contents, monosaccharides distributions and impurities (including protein, phenolics and uronic acids). *E. maxima* and *E. radiata* fucoidans contained high amounts of total carbohydrates, with 72.8% and 88% (*w*/*w*), respectively (Table 1). The *S. elegans* and *F. vesiculosus* fucoidans had approximately 40% (*w*/*w*) total carbohydrate content (Table 1) and were, therefore, comparatively lower than that of the *Ecklonia* sp. extracted fucoidans.

After hydrolysing the fucoidans using 2 M TFA, monosaccharides were quantified using HPLC and Megazyme kits (Table 1). The predominant monosaccharides detected in all fucoidans were fucose, glucose, galactose, and mannose. Generally, *Ecklonia* sp. fucoidans had a relatively high monosaccharide content, with glucose, fucose, galactose and mannose being the most prominent sugars (Table 1). These findings are consistent with the findings of January and colleagues, who detected considerable amounts of glucose, galactose, and mannose in their *E. maxima* fucoidan extract [37]. The commercial *F. vesiculosus* fucoidan contained relatively higher levels of fucose than that of the extracted fucoidans (Table 1). Furthermore, *S. elegans* fucoidan had notably higher mannose content than the other fucoidans (Table 1). The monosaccharide distribution of the extracts is representative of the characteristic fucoidans.

Commercial *F. vesiculosus* had the highest sulphate content (about 15%), followed by *S. elegans* fucoidan, which had 9.7% (Table 1). *S. elegans* and *F. vesiculosus* fucoidans had higher sulphate contents than the *Ecklonia* sp.-derived fucoidans (between 7–8%). The higher sulphates within *F. vesiculosus* and *S. elegans* fucoidan determined by colourimetry agreed with the structural characterisation data (Figure 1 and Figure 2). FTIR spectra at wavenumber around 845 cm^−1^ showed a more pronounced peak for the *F. vesiculosus* fucoidan than all extracts (Figure 1). The pronounced NMR peaks indicative of sulphates between ppm 1.2 and 1.6 were evident for the *F. vesiculosus* and *S. elegans* than for *Ecklonia* sp. fucoidans (Figure 2). Additionally, the ash content was higher within the *S. elegans* and *F. vesiculosus* fucoidans (Table 1), suggesting more sulphates among these fucoidans than the *Ecklonia* sp. fucoidans. The ash content detected from the fucoidans was between 19 and 24%, consistent with the ash contents in some characterised fucoidans [15,38]. Furthermore, the fucoidans had minimal protein and uronic acid contamination, with *S. elegans* having the highest at ~4% of each. Insignificant amounts (<2%) of phenolics were detected within the fucoidans (Table 1). The molecular weights of the fucoidans were determined by size exclusion HPLC. The molecular size of *E. maxima* fucoidan was 27.4 kDa, *E. radiata* fucoidan was 8.5 kDa, *S. elegans* fucoidan was 74.9 kDa, and *F. vesiculosus* fucoidan was 84.4 kDa. Structural and chemical characterisation data suggest that the extracted crude fucoidans were relatively pure as they showed similar profiles to the commercial *F. vesiculosus* fucoidan and other previously characterised fucoidans in literature. 

### 2.4. Fucoidans’ Cytotoxicity to HCT116 Cancer Cells

**Figure 4:**
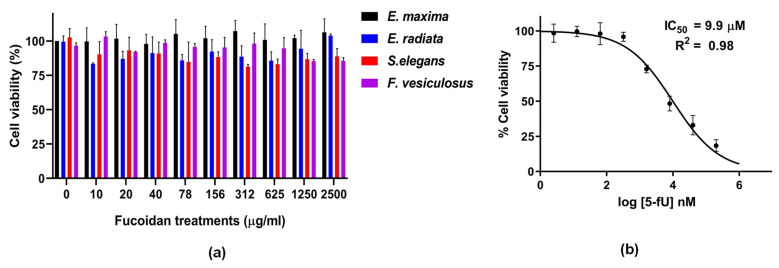
Fucoidans’ cytotoxicity on HCT116 cells assessed by the resazurin assay. (**a**) Cell viability after treatment with fucoidans, (**b**) IC_50_ curve of 5-FU (positive control) demonstrates the compound’s cytotoxic effect on HCT116 cells. The data represent values obtained from 3 biological replicates expressed as means ± SD (*n* = 3).

The potential cytotoxicity of all the fucoidan extracts towards the HCT116 colon cancer cell line was examined and compared to the chemotherapeutic drug 5-fluorouracil (5FU). The colon cancer cell line HCT116 was selected with the probable oral route of administration of fucoidans. For decades 5FU has played a pivotal role in the treatment of colorectal cancer [39]. Thus, it was chosen as a positive control for our experiments. The 5-FU treatments showed robust anti-cancer activity with an IC_50_ value of 9.9 µM. The reduction in cell viability due to treatment with fucoidan extracts was expressed as the percentage of viable cells remaining after treatment compared to the vehicle-treated control cells. Even at 2.5 mg/mL loading, none of the fucoidans displayed any significant cytotoxic effect on the HCT116 cells (Figure 4). About 4 g/day of fucoidan has been used in combination with other chemotherapeutics, including 5-FU, in colorectal cancer patients. Although patient survival was improved when fucoidan was included in the treatment, a significant observation was reduced side effects [40]. 

The lack of cytotoxicity of the fucoidans could be attributed to the large molecular sizes (Table 1), making penetration into the cells difficult. Large molecular sizes of fucoidans have been reported to limit the bio-accessibility of these compounds, posing a challenge for their applications [41]. Native *Undaria pinnatifida* fucoidan had minimal anti-tumour activity compared to its depolymerised counterpart against the human lung cancer cell line A549 [42]. This observation suggests a need for depolymerising fucoidans to increase toxicity while at the same time maintaining their bioactivities. We acknowledge that size cannot be the only determining factor, but other fucoidans’ characteristic factors, including sulphation, and monosaccharide distribution, will contribute to their bioactivities.

#### 2.4.1. The Effect of Fucoidans on HCT116 Colony Formation

**Figure 5:**
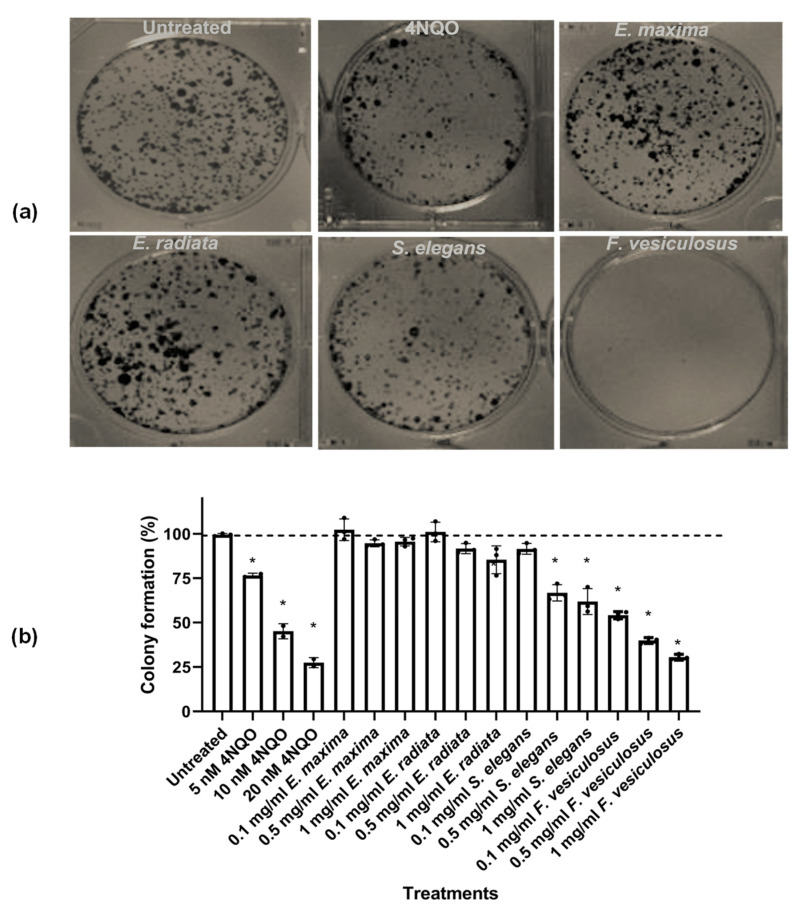
The dose-dependent clonogenic effect of fucoidan on HCT116 cancer cells. (**a**) Visual representation of the effect of fucoidan extracts (1 mg/mL) on HCT116 colony formation; (**b**) Dose-dependent effect of compounds on HCT116 cells’ colony formation. The HCT116 colony cells were calculated and expressed as the means ± SD percentages (*n* = 3). The * shows a significant treatment difference versus the untreated control (*p* < 0.05) analysed by One-way ANOVA.

Having established that fucoidans did not show substantial cytotoxicity, these compounds were further tested for their ability to inhibit colony formation. This assay has been the method of choice to determine replicative cell death after ionising radiation, although it is also used to determine the effectiveness of other cytotoxic agents [43]. *S. elegans* and *F. vesiculosus* fucoidans were significant inhibitors of HCT116 colony formation (*p* < 0.05) (Figure 5). The positive control 4-NQO was used in this assay and showed the dose-dependent inhibition of HCT116 cell colony formation. 

The *S. elegans* fucoidan exhibited about 40% colony formation inhibition at 0.5 mg/mL. The *F. vesiculosus* fucoidan inhibited HCT116 cell colony formation by over 50% at 0.1 mg/mL concentration (Figure 5). The inhibition by *F. vesiculosus* and *S. elegans* fucoidans may be attributed to the superior sulphate content compared to that of the *Ecklonia* sp. derived fucoidans (Table 1). A limited number of studies have reported the ability of fucoidans to decrease tumour cell survival using this assay. Nevertheless, our findings agree with those of Shin and colleagues, who reported that manganese dioxide nanoparticles coated with fucoidan decreased colony formation by a pancreatic cancer cell line [44]. Another independent study reported fucoidan inhibited colony formation of HepG2 liver cancer cells [20]. 

#### 2.4.2. Fucoidans Inhibit the 2D Migration of HCT116 Cancer Cells

**Figure 6:**
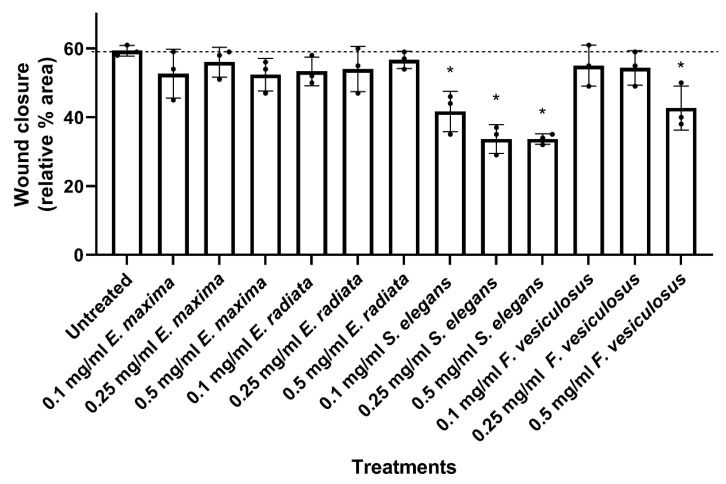
The effect of fucoidan extracts on 2D HCT116 cell migration. Quantified migration profiles of HCT116 cells treated with *E. maxima, E. radiata, S. elegans* and *F. vesiculosus* fucoidan extracts relative to the untreated control. The data are represented as means ± SD of biological replicates (*n* = 3). The asterisk * represents treatment concentrations that had a statistically significant effect on the migration of the cells at *p* < 0.05 tested by One-way ANOVA.

Fucoidans were next tested for effects on the 2-dimensional (2D) migration of human HCT116 colorectal cancer cells using the wound healing assay. The *F. vesiculosus* and *S. elegans* fucoidans significantly inhibited cell migration compared to the untreated control (Figure 6). *S. elegans* fucoidan showed a dose-dependent inhibition of HCT116 cell migration at all concentrations tested, with inhibition reaching up to 30% at about 0.25 mg/mL (Figure 6), while cell migration inhibition by fucoidan from *F. vesiculosus* was only significant at 0.5 mg/mL.

The *Ecklonia* sp. fucoidans did not significantly inhibit HCT116 cancer cell migration, even at high concentrations (Figure 6). This observation may be linked to the high amount of sugars within their structure (Table 1). Literature has suggested that fucoidans consisting of sugars, including galactose, may provide the nutrition required for wound healing [45]. However, fucoidans with a higher sulphate concentration were associated with a better bioactivity [36,46]. Thus, we can infer that *S. elegans* fucoidan showed better inhibitory action to wound healing of the HCT116 cells due to its unique structural properties, including high sulphation (Figure 1 and Figure 2).

#### 2.4.3. Fucoidans Inhibit HCT116 3D Spheroid Migration

**Figure 7:**
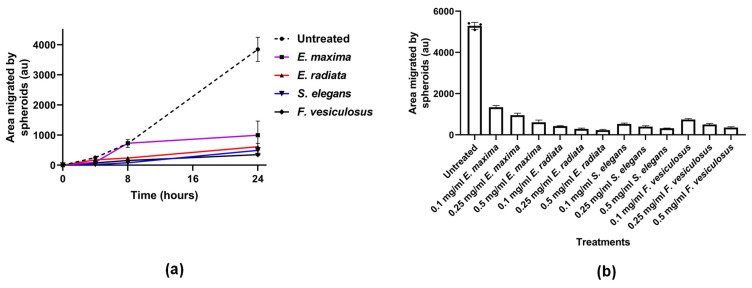
The effect of fucoidans on the 3D HCT116 spheroid migration. (**a**) Time-dependent effect of fucoidans at a fixed concentration (0.1 mg/mL) on spheroid migration; (**b**) Quantification of the dose-dependent effect of fucoidans on 3D spheroid HCT116 migration. The data are represented as means ± SD of biological replicates of spheroids (*n* = 3). One-way ANOVA was used to compare treatments to the untreated experiments, where significance was considered at *p* < 0.05. No asterisks * are shown since all treatments differed significantly from the untreated experiments.

Next, we tested the ability of fucoidans to prevent the migration of cells from a three-dimensional sphere onto tissue culture plastics. In this assay, the fucoidans inhibited the migration of the HCT116 cells from spheres in a time-dependent manner (Figure 7a). The commercial *F. vesiculosus*, *S. elegans* and *E. radiata* fucoidans displayed comparable efficacies, showing more than 80% inhibition at 0.1 mg/mL concentration. Furthermore, inhibition of HCT116 spheroid migration by the fucoidans was dose-dependent (Figure 7b). Although *E. maxima* fucoidan showed a slightly lower inhibition potential than the other fucoidans, it still significantly (*p* < 0.05) inhibited migration from HCT116 spheres (Figure 7b). 

Notably, the fucoidan extracts showed potency in inhibiting HCT116 cell migration during time- and dose-dependent experiments. The anionic nature, which is the common characteristic of fucoidans, could be critical in disrupting the migration of HCT116 cells. Limited reports in the literature have investigated the effects of fucoidans on spheroid-based migration. However, a study by Han and colleagues showed that tumour migration of a human colon cancer cell line (HT-29) was inhibited by fucoidan [17]. Indeed, very few investigations on the potency of chemotherapeutics on spheroid migration have been reported [47]. In addition, spheroid culture systems provide similar physicochemical environments to in vivo models, making them ideal for studying tumour migration—however, their use in fucoidan studies is seldom reported. The fucoidans in the current study demonstrated their high potency in inhibiting 3D HCT116 migration from spheroids, which may be important in controlling the proliferation of colorectal cancers. Another merit of employing spheroid culture systems is that they involve cell-to-cell and cell-to-matrix interactions, which overcomes the limitations of traditional monolayer cell cultures, which are two-dimensional (2D) [47,48]. Fucoidans maybe be interfering with cell-to-matrix adhesion or even with cell-to-cell interactions.

#### 2.4.4. Fucoidans Disrupt Cancer Cell Sphere Formation

**Figure 8:**
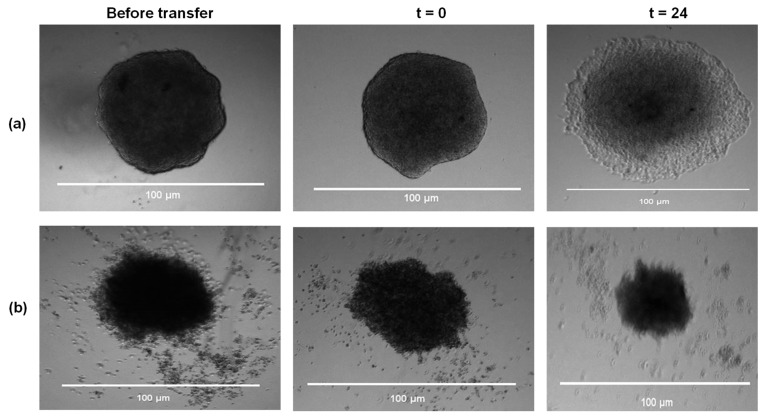
A representative visual illustration of HCT116 spheroid pretreated with fucoidan. The images show spheroids before transfer to fresh medium, at t = 0, and after 24 h (t = 24). (**a**) representative sphere formed from an untreated HCT116 culture; (**b**) sphere formed from HCT116 cells pretreated with *F. vesiculosus* fucoidan at 0.5 mg/mL final concentration.

Next, the HCT116 cells were pretreated with 0.1 mg/mL and 0.5 mg/mL of fucoidans to determine whether fucoidans inhibit sphere formation. Representative morphological data of the HCT116 cell spheres pretreated with 0.5 mg/mL of *F. vesiculosus* illustrated a common observation for all fucoidans tested (Figure 8). Fucoidan treatment disrupted the formation of spheres compared to those from untreated samples (Figure 8). 

**Figure 9:**
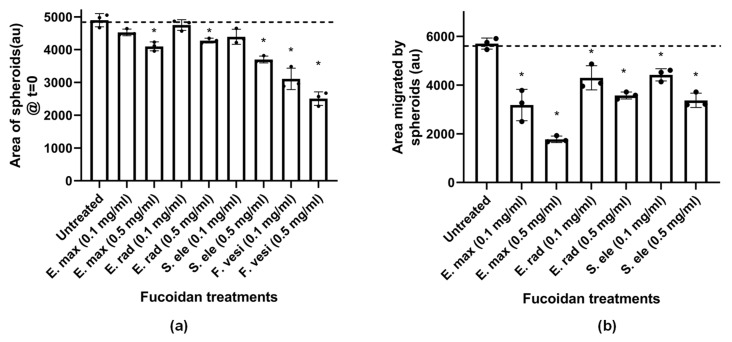
Fucoidans hinder HCT116 spheroid formation and reduce migration from spheres. (**a**) Size of HCT116 spheroids; (**b**) Distance migrated on tissue culture plastic from pretreated spheroids. The data are represented as means ± SD of biological replicates of spheroids sizes and migration (*n* = 3). The asterisk * represents treatment concentrations that were statistically significant from the untreated cells at *p* < 0.05 tested using One-way ANOVA.

The HCT116 sphere sizes formed after pre-treatment with fucoidans were quantified (Figure 9a). All the fucoidans significantly reduced the size of spheroids formed compared to the untreated sample (Figure 9a; *p* < 0.05).

The pretreated spheroids were subsequently transferred to an untreated medium to investigate the migration of cells from the spheres back onto tissue culture plastic (Figure 9b). Interestingly, all the spheres pretreated with fucoidan showed reduced migration compared to untreated spheroids (Figure 9b; *p* < 0.05). Therefore, pretreatment of the HCT116 cell culture indicated that fucoidans hindered spheroid formation and subsequent migration onto the tissue culture plastic matrix. In addition, the spheres which were pretreated with *F. vesiculosus* fucoidan were distorted and failed to migrate. Although investigations on spheroid formation are largely unexplored as far as the use of fucoidans is concerned, Han and colleagues reported that *F. vesiculosus* fucoidan disrupted HT-29 spheroid formation [19]. Their findings concur with our sphere formation results (Figure 8). Although this technique is a useful tool, it is limited to very few in vitro studies. However, our findings can be used as a motivation to further pursue the potential of fucoidans in in vivo and clinical settings.

#### 2.4.5. Fucoidans Inhibit HCT116 Cell Adhesion

**Figure 10:**
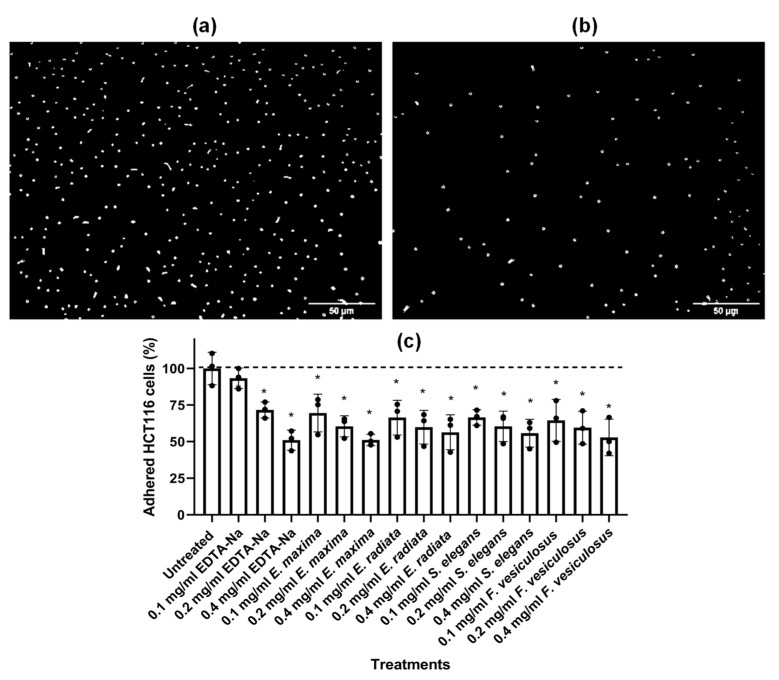
Fucoidan inhibits the adhesion of HCT116 cancer cells. (**a**) untreated cells; (**b**) cells treated with fucoidan under light microscopy; (**c**) Quantification of HCT116 cancer cells adhesion by crystal violet. The data are represented as means ± SD of three biological replicates (*n* = 3). The asterisk * represents treatment concentrations that were statistically significant from the untreated cells at *p* < 0.05 tested using One-way ANOVA.

The effect of fucoidan extracts on HCT116 cell adhesion was also investigated. The fucoidans significantly prevented the adhesion of HCT116 cells to tissue culture plastic (Figure 10).

The dose-dependent inhibition of HCT116 cancer cell adhesion by fucoidan was quantified by crystal violet (Figure 10c). EDTA-Na, a known chelator of metal ions required for cell adhesion, was used as a positive control. All fucoidans were efficient inhibitors of cell adhesion. Cell adhesion within cancer cells is vital for various biological processes, including cellular organisation, communication, differentiation, migration, and metastasis [49]. The cancer cell adhesion is dependent on several adhesion molecules and receptors, including integrins, selectins, glycoproteins, and proteoglycans [49]. The fucoidans may have hindered the proper functioning of these molecules, thereby impacting the adhesion of cancerous cells. Some fucoidans prevent the adhesion of cancer cells onto the extracellular matrix (ECM). Fucoidan from *A. nodosum* inhibited the MDA-MB-231 cancerous cells adhering to fibronectin ECM [50], consistent with our findings on tissue culture plastic. Fucoidans are negatively charged polysaccharides due to their sulphated nature, which may interfere with integrins that require Mg^2+^ as a cofactor for adhesion [49]. Thus, it is possible to suggest our fucoidans inhibited the HCT116 cancer cells’ adhesion in a similar mechanism. This observed effect of fucoidans on cell adhesion might also explain the effect of these compounds in inhibiting the formation and migration from spheres (Figure 8 and Figure 9). However, HCT116 cells’ adhesion cannot be the only process affected by fucoidans, as *E. maxima* and *S. elegans* extracts show similar anti-adhesion properties but show radically different effects in the colony formation assay (Figure 5). A complex combination of structural characteristics, including the degree of sulphation, molecular size, and carbohydrate content, should be essential to fucoidans’ biological activities. The observed anticancer activities of fucoidans may be useful as a preventive/treatment strategy for CRC since they are likely to be administered orally. 

## 3. Materials and Methods

*Fucus vesiculosus* fucoidan (Cat. No. F5631) was purchased from Sigma-Aldrich (St. Louis, MO, USA). The analytical kits used in this study were purchased from Megazyme^TM^ (Bray, WC, Ireland). The other reagents were purchased from Sigma-Aldrich, MERCK, Flucka Saarchem (Darmstadt, HE, Germany), and Celtic Diagnostic and Life Technologies (Cape Town, South Africa).

### 3.1. Sampling and Seaweed Processing

The brown seaweeds, *Ecklonia radiata* and *Sargassum elegans,* were harvested between February and March 2019 from Kelly’s beach in Port Alfred (coordinates 33°36′36.8424″ S; 26°53′23.4996″ E) in the Eastern Cape province, South Africa. *Ecklonia maxima* seaweed was kindly donated by the HIK-Abalone farm located in Hermanus, Western Cape province, South Africa. Most of the *E. radiata* seaweed was collected as beach cast. However, some were harvested together with the *S. elegans* from rock pools. The beach cast and rockpool collected *E. radiata* were mixed and processed as a single batch. The harvested seaweeds were stored on ice during transportation to the laboratory. Upon arrival at the laboratory, the seaweed was washed 3× with distilled water, cut into smaller pieces and oven-dried at 40 °C for 72 h. The dried seaweed was pulverised using a coffee grinder, and the resulting powder was stored at room temperature until use.

### 3.2. Hot Water Extraction

The seaweeds were defatted, and pigments were extracted using a high methanol percentage mixture, with a solvent ratio of 4:2:1 for MeOH: CHCl_3_: H_2_O [51,52]. The fucoidans were hot water extracted as described by Lee and co-workers with minor modifications [53]. A mass of 15 g dry defatted seaweed powder was suspended in 450 mL of distilled water in a ratio of 1:30 (*w*/*v*). The mixture was heated to 70 °C with agitation overnight. The extracted fucoidan yield was expressed as a percentage of the dry defatted seaweed weight (% dry wt).

### 3.3. Structural Validation of Extracted Fucoidans

#### 3.3.1. Fourier Transform Infrared Spectrometry (FTIR) Analysis

A hundred milligrams of ground fucoidan was scanned using Fourier-transform infrared spectroscopy (FTIR) on a 100 FT-IR spectrometer system (Perkin Elmer, Wellesley, MA, USA). The signals were automatically recorded by averaging four scans over 4000–650 cm^−1^. The baseline and ATR corrections for penetration depth and frequency variations were performed using Spectrum One software (version 1.2.1) (Perkin Elmer, Wellesley, MA, USA).

#### 3.3.2. NMR Spectroscopy Analysis

Fucoidan samples (10 mg) were dissolved in 1 mL D_2_O. After centrifugation at 13,000× *g* for 2 min, any insoluble matter was removed by filtering the supernatant through a 0.45-μm filter. The deuterium-exchanged samples were subjected to ^1^H-NMR analysis, and spectra were recorded at 23 °C using a 400 MHz spectrometer (Bruker, Fällanden, Switzerland) with Topspin 3.5 software (Bruker, Billerica, MA, USA). 

#### 3.3.3. Thermogravimetric Analysis

Fucoidans were subjected to thermogravimetric analysis using a Pyris Diamond model thermogravimetric analyser (PerkinElmer^®^, Shelton, CT, USA). Samples of 4 mg fucoidan were analysed in an aluminium crucible. Pure nitrogen (purity of 99.99%) was used as the carrier gas during all the experiments to reduce the mass transfer effect. The gas flow rate was at 20 mL/min. The fucoidans were heated from 30 °C to 900 °C at a heating rate of 30 °C/min. A separate blank using an empty tray was run for baseline correction. Lastly, the mass loss relative to the temperature increment was automatically recorded, and the derivative thermogram (DTG) was plotted using GraphPad Prism version 6.

### 3.4. Chemical Characterisation of Fucoidans

Using *L*-fucose as a standard, the phenol-sulphuric acid method estimated the total sugar content within the fucoidans [54]. The total reducing sugar content in 2 M TFA partially hydrolysed fucoidans was quantified using the dinitrosalicylic acid (DNS) assay [55]. Furthermore, the protein contamination was measured using Bradford’s method, utilising bovine serum albumin (BSA) as a standard [56]. The sulphate content in formic acid (60% *v*/*v*) desulphurised fucoidan was measured using a barium chloride–gelatin method as described previously [57], which was scaled down to microtitre volumes. 

Polyphenols within the fucoidans were quantified using a modified Folin–Ciocalteu method with gallic acid as a standard [58]. Moreover, quantitative analyses of *L*-fucose, *d*-fructose, *d*-galactose, *d*-xylose, *l*-arabinose, and *d*-mannose in the fucoidans were performed using high-performance liquid chromatography (HPLC) method [16]. A Shimadzu HPLC (RID) instrument (Kyoto, Japan) and a Fortis Amino column (Fortis Technologies Ltd., Cheshire, UK) was utilised in the HPLC method. The ash contents in fucoidans were derived from derivative thermogravimetry (DTG) data. 

### 3.5. Determination of Fucoidans Molecular Weights by HPLC

The molecular weights of fucoidans were determined using size exclusion high-performance liquid chromatography with a refractive index detector (HPLC-RID). The fucoidan extracts were separated using a Shodex OHpak SB-806M HQ (8.0 mm I.D. × 300 mm) column (Showa Denko, Tokyo, Japan) according to the manufacturer’s recommendations. The mobile phase (0.1 M NaNO_3_ aq) used was filtered through 0.22 µm nylon membranes (Membrane solutions, Auburn, USA). The flow rate was adjusted to 0.6 mL/min, the column temperature was at 30 °C, and the sample injection volume was 20 µL. Pullulan standards (Shodex, Tokyo, Japan) were used to construct the standard curve for interpolating fucoidan molecular weights.

### 3.6. Cell Culture

The HCT116 human colon cancer cell line was from the American Type Culture Collection (ATCC CCL-247). The cell line was cultured in Dulbecco’s Modified Eagle’s Medium (DMEM) with GlutaMAX™-I, supplemented with 10% (*v*/*v*) fetal bovine serum (FBS) and 1% (*v*/*v*) sodium pyruvate. The cell culture was maintained at 9% CO_2_ in a humidified incubator at 37 °C.

### 3.7. Cytotoxicity Screening 

The susceptibility of the HCT116 cell line to the fucoidan extracts was determined using an optimised resazurin assay [16]. Briefly, cells were seeded at a density of 1 × 10^5^ cells/well in DMEM growth medium in a 96-well plate. After the cells adhered to the plate matrix overnight, they were treated with varying doses of fucoidan (0.1 mg/mL to 2.5 mg/mL). The anti-cancer agent 5-fluorouracil (5-FU) in a concentration range of 0.0064 µM to 2500 µM was included as a positive control for cytotoxicity. The experiments were incubated for 72 h before treatment with resazurin. Cell viability was measured by fluorescence (excitation = 560 nm and emission = 590 nm). 

### 3.8. Clonogenic Assay

HCT116 cells were seeded at a density of 1.5 × 10^3^ cells/mL in a six-well plate and allowed to adhere overnight. The cells were treated with fucoidan extracts or 4-nitroquinoline 1-oxide (4NQO), which was used as a positive control. The cultures were incubated at 37 °C for 48 h, upon which half the volume of spent medium was removed and replaced with fresh medium lacking treatment. The cultures were incubated, and the medium changed every two days until individual colonies of at least 50 cells/colony were visible. The medium was removed, and the cells were washed once with PBS. The cells were fixed for 10 min by a 3:1 methanol to the acetic acid mixture. The fixative was removed, and the plate was allowed to air dry for 2 min. The HCT116 cell colonies were stained with 5% (*w*/*v*) crystal violet in methanol for 4–6 h, washed three times in PBS and rinsed in water. The plates were air-dried, and the images were captured using a ChemiDoc^™^-XRS (BioRad, Hercules, CA, USA). The cells were solubilised completely using 1 M acetic acid, and the absorbance was read at a wavelength of 590 nm. The % colony formation was expressed as percentiles relative to the untreated experiments.

### 3.9. Wound Healing Assay

A volume of 500 µL/well of HCT116 cells were seeded at 7 × 10^5^ cells/mL into 24 well plates. The cells were allowed to adhere and grow to 100% confluence overnight at 37 °C. A wound was made down the centre of the well with a pipette tip. After wounding, the floating cells were removed, and fresh medium without or with varying doses of fucoidan (0.1–0.5 mg/mL) treatments was added. Pre-migration images of the wounds were taken at 4× magnification. The plates were further incubated at 37 °C for 12 h, whereafter, images were taken at the same position as the premigration images. The images were analysed on ImageJ using a wound healing plugin [59], and wound closure was calculated using the formula below where the percentage wound area was calculated relative to the wound size at t = 0:

$$\mathrm{Wound}\;\mathrm{closure}=\%\mathrm{wound}\;\mathrm{area}\left(t=0\right)-\%\mathrm{wound}\;\mathrm{area}\;(t=12)$$

### 3.10. Sphere-Based Tumour Migration Assays

HCT116 cells were resuspended in an appropriate volume of Dulbecco’s Modified Eagle’s Medium (DMEM) (final concentration of 1 × 10^4^ cells/10 µL) for the formation of spheres in an optimised hanging drop method [60]. About 5 mL of sterile PBS was pipetted into the bottom portion of the tissue culture plate (100 mm diameter) to create a humidified environment., Multiple 10 µL culture drops were deposited inside the lid of the culture dish. The lid with the hanging drops was placed back on top of the PBS-containing dish, taking care to avoid disturbing the droplets. The plate was incubated for 48 h at 37 °C to allow the spheres to grow. The spheres were then transferred to a 24-well plate prefilled with 300 µL medium and respective treatments with compounds ranging from 0.1 mg/mL to 0.5 mg/mL. In some experiments, the HCT116 culture (at a density of 10,000 cells/10 µL) was pretreated with 0.5 mg/mL and 0.1 mg/mL of the compounds during sphere formation. Untreated or pretreated spheres transferred to the adherent plate were allowed to adhere by incubation at 37 °C for 4 h. Images were taken at 4× magnification and this time was taken as t = 0 (4 h post-seeding of spheres into adherent plates). The spheroids were monitored over time for t = 24 h. The areas of migration were quantified using Fiji/ImageJ (Version 1.53f51). The results presented were represented by three experimental biological replicates. The data were normalised to the initial size of each spheroid at time 0 to determine cell migration from the spheroid. The migration of cells from spheres was calculated as follows:

Distance migrated=area measured at t=24 hrs−area at t=0 h

### 3.11. Cell Adhesion Assays

HCT116 cells were seeded at a density of 6 × 10^4^ cells/well in a 96-well plate and treated with varying concentrations of fucoidan (0.1–0.4 mg/mL) or left untreated as the control. The culture was incubated at 37 °C with 9% CO_2_ for 8 h. The spent medium was decanted from the plate, and the adhered cells were washed thrice with 1 × sterile PBS (137 mM NaCl, 2.7 mM KCl, 10 mM Na_2_HPO_4_ and 2 mM KH_2_PO_4_, pH = 7). The cells were fixed by adding 100 µL/well of a 3:1 methanol: acetic acid solution and incubated at room temperature for 15 min. The methanol-acetic acid mixture was washed off using sterile distilled H_2_O and blotted dry on a paper towel. The cells were stained with 0.1% (*w*/*v*) crystal violet dye (40 µL/well) and incubated at room temperature for 20 min with gentle agitation at 30 rpm. The crystal violet dye was discarded, and the plate was washed four times with distilled water. A volume of 100 µL/well of 1% (*w*/*v*) SDS was added, the plate incubated overnight, and the optical density was then measured at a wavelength of 590 nm. 

## 4. Conclusions

The extracted compounds were unique in composition, with *Ecklonia* sp. fucoidans having a relatively high carbohydrate content compared to *S. elegans* and commercially purchased *F. vesiculosus*. Furthermore, the *Ecklonia* sp. fucoidans had a comparatively low degree of sulphation compared to the other fucoidans, despite having comparatively lower molecular weights. Their low molecular weight could have had an impact on the anti-adhesion and anti-spheroid migration of the HCT116 cancer cells. Although slightly larger, the *S. elegans* and *F. vesiculosus* fucoidans had a relatively higher sulphate content than the *Ecklonia* sp. fucoidans, which may have enhanced the anti-cancer activities of these fucoidans observed by the anti-colony formation, anti-adhesion and anti-spheroid migration of the HCT116 cells. Our study reaffirms that molecular weight and sulphation of fucoidan, along with other properties, may be important for biological activity. This study also showed that fucoidans differ in structure and activity depending on the type (genus and species) of seaweed. Although there are structural differences between the fucoidans studied, their anti-cancer effects suggest some potential health benefits of seaweed fucoidans that warrant further analysis. 

## Figures and Tables

**Table 1:** Composition of fucoidan structures.

	*w*/*w* % ± SD			
Component	*E. maxima*	*E. radiata*	*S. elegans*	*F. vesiculosus*
Total carbohydrates ^a^	72.8 ± 5.2	88 ± 7.4	44.4 ± 6.2	41.3 ± 9.5
*L*-fucose ^b^	4.56 ± 0.8	3.7 ± 0.1	4.9 ± 0.9	8.2 ± 0.4
*D*-glucose ^b^	8.1 ± 3.4	7.1 ± 2.3	5.7 ± 1.7	5.1 ± 2.1
*D*-galactose ^b^	4.8 ± 0.1	4.9 ± 1.2	5.7 ± 0.5	7.1 ± 1.83
*D*-mannose ^b^	3.0 ± 0.5	4.2 ± 0.2	7.1 ± 1.8	4.5 ± 0.8
Total sulphates ^c^	7.2 ± 1.2	8.8 ± 1.4	9.7 ± 1.8	14.7 ± 2.3
Total phenolics ^d^	1.9 ± 0.6	1.9 ± 0.4	2.8 ± 0.8	0 ± 0.04
Uronic acids ^e^	2.6 ± 1.2	2.2 ± 0.7	4.8 ± 0.6	2.2 ± 0.8
Total protein ^f^	2.1 ± 0.6	2.4 ± 0.9	4.6 ± 2.4	1.9 ± 0.6
Total ash ^g^	19.7 ± 0.6	16.0 ± 2.1	23.1 ± 3.5	20.4 ± 2.8
MW ^h^	27.4 kDa	8.5 kDa	74.9 kDa	84.4 kDa

Determined by ^a^ Phenol sulphuric acid method; ^b^ HPLC (RID); ^c^ Barium chloride gelatin method; ^d^ Folin-Ciocalteu method; ^e^ MegazymeTM uronic acid kit; ^f^ Bradford’s assay; ^g^ TGA; ^h^ size exclusion HPLC.

## Data Availability

Data are available upon request.
